# Minor contribution of ammonia oxidizers to inorganic carbon fixation in the ocean

**DOI:** 10.1038/s41561-025-01798-x

**Published:** 2025-09-23

**Authors:** Barbara Bayer, Katharina Kitzinger, Nicola L. Paul, Justine B. Albers, Mak A. Saito, Michael Wagner, Craig A. Carlson, Alyson E. Santoro

**Affiliations:** 1 Centre for Microbiology and Environmental Systems Science, Department of Microbiology and Ecosystem Science, University of Vienna, Vienna, Austria; 2 Department of Ecology, Evolution and Marine Biology, University of California, Santa Barbara, Santa Barbara, CA USA; 3 Marine Chemistry and Geochemistry Department, Woods Hole Oceanographic Institution, Woods Hole, MA USA; 4 Center for Microbial Communities, Department of Chemistry and Bioscience, Aalborg University, Aalborg, Denmark

**Keywords:** Microbial ecology, Carbon cycle, Microbial biooceanography

## Abstract

Ammonia-oxidizing archaea are the most abundant chemolithoautotrophs in the ocean and are assumed to dominate carbon fixation below the sunlit surface layer. However, the supply of reduced nitrogen delivered from the surface in sinking particulate organic matter is insufficient to support the amount of nitrification required to sustain measured carbon fixation rates in the dark ocean. Here we attempt to reconcile this observed discrepancy by quantifying the contribution of ammonia oxidizers to dark carbon fixation in the eastern tropical and subtropical Pacific Ocean. We used phenylacetylene—a specific inhibitor of the ammonia monooxygenase enzyme—to selectively inhibit ammonia oxidizers in samples collected throughout the water column (60–600 m depth). We show that, despite their high abundances, ammonia oxidizers contribute only a small fraction to dark carbon fixation, accounting for 4–25% of the total depth-integrated rates in the eastern tropical Pacific. The highest contributions were observed within the upper mesopelagic zone (120–175 m depth), where ammonia oxidation could account for ~50% of dark carbon fixation at some stations. Our results challenge the current view that carbon fixation in the dark ocean is primarily sustained by nitrification and suggest that other microbial metabolisms, including heterotrophy, might play a larger role than previously assumed.

## Abstract (web summary)

Ammonia oxidizers contribute minimally to carbon fixation in the dark ocean despite their abundance at depth, according to enzyme inhibition experiments on water samples from two cruises.

## Main

Phytoplankton-driven primary production is one of the most important biological processes converting dissolved inorganic carbon (DIC) into organic matter, forming the base of the marine food web^1^. While most of the DIC fixation in the surface ocean is fuelled by light energy, non-photosynthetic DIC fixation (‘dark DIC fixation’) has been proposed to contribute substantially to the biological uptake of DIC in the ocean^2,3^, possibly increasing global ocean primary production estimates by up to 22% (refs. ^4,5^). In surface waters, dark DIC fixation has primarily been associated with the activities of heterotrophic bacteria^6,7^, which can incorporate DIC into biomass via various carboxylation reactions involved in central metabolic functions such as C assimilation, anaplerosis and/or redox-balancing^8,9^.

Below the sunlit surface layer, the downward flux of particulate organic material is considered to be the main source of organic C sustaining the dark ocean’s heterotrophic food web^10^. However, current estimates point to a mismatch between organic matter consumption and supply in the deep ocean^11,12^, implying that additional sources of organic C are required to reconcile the C budget in the mesopelagic zone (defined here as below the euphotic zone to 1,000 m depth)^13,14^. DIC fixation rates can be substantial in the dark ocean^13,15^ and are often comparable in magnitude to microbial heterotrophic activity^13,16,17^. In aphotic waters, DIC fixation has largely been attributed to the activities of chemolithoautotrophic bacteria and archaea that use the energy released from oxidizing reduced compounds to fuel a variety of inorganic C fixation pathways^13,18–20^. Chemolithoautotrophic production is a source of particulate organic C that could contribute substantially to the microbial heterotrophic C demand in the dark ocean^13,15,21^. However, the activities of chemolithoautotrophic microorganisms are typically not well accounted for in mesopelagic C budgets^22,23^. An improved understanding of microbial processes in the mesopelagic zone is essential to better predict C export and sequestration, particularly under future climate change scenarios^24,25^.

Organic matter remineralization releases ammonium^26^, which is the primary energy source for chemolithoautotrophy in most parts of the global ocean^18^. Consequently, nitrification (the microbial oxidation of ammonia (NH_3_) to nitrite (NO_2_^−^) and further to nitrate (NO_3_^−^)) is expected to be tightly linked to dark DIC fixation. However, dark DIC fixation and nitrification are not routinely measured on oceanographic expeditions—particularly not in combination—making it impossible to accurately infer such a relationship. Empirical conversion factors obtained from pure culture studies are often used to estimate dark DIC fixation rates from nitrification rates^27–29^ and vice versa^13,30^. Measurements of DIC fixation in the deep ocean are on average one order of magnitude higher than could be supported by ammonium supplied by the sinking flux of particulate organic nitrogen (N) according to estimates of global ocean N export^18,27,28^. Multiple hypotheses have been proposed to explain this large discrepancy, including: (1) unaccounted sources of ammonium to the deep ocean; (2) energy sources other than ammonia that support chemolithoautotrophy; and (3) heterotrophic microorganisms being a major contributor to dark DIC fixation^14^. However, direct evidence supporting any of these scenarios is thus far lacking.

In this study we established a methodological framework to specifically inhibit ammonia-oxidizing microorganisms in ocean samples. We then quantified the fraction of dark DIC fixation fuelled by ammonia oxidation throughout the water column of the eastern Pacific Ocean. Finally, we explored the relationships between dark DIC fixation, ammonia oxidation and heterotrophic production. The results of this study help to reconcile the observed discrepancies between N supply and DIC fixation at depth, and advance our understanding of microbial processes in the dark ocean.

## Environmental context

**Fig. 1:**
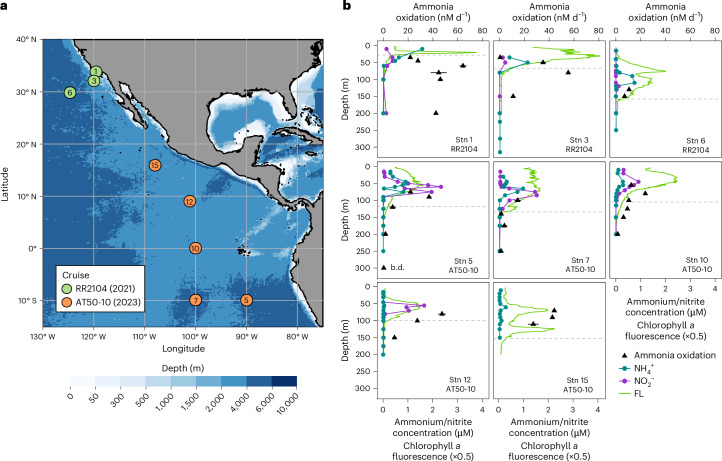
Cruise tracks and environmental context. **a**, Station map of cruises RR2104 (green) and AT50-10 (orange). Bathymetry data from ref. ^58^. **b**, Ammonia oxidation rates, chlorophyll *a* fluorescence (FL) and ammonium (NH_4_^+^) and nitrite (NO_2_^−^) concentrations at eight stations during cruises AT50-10 and RR2104. NO_2_^−^ concentrations were not measured at Stn 15. For ammonia oxidation rates, the mean and s.d. (error bars) of three biological replicates are shown (b.d., below the calculated detection limit of the method). Note that the error bars for ammonia oxidation rates are often smaller than the symbols. The depth of the euphotic zone is indicated by the grey dashed horizontal lines.

**Extended Data Fig. 1:**
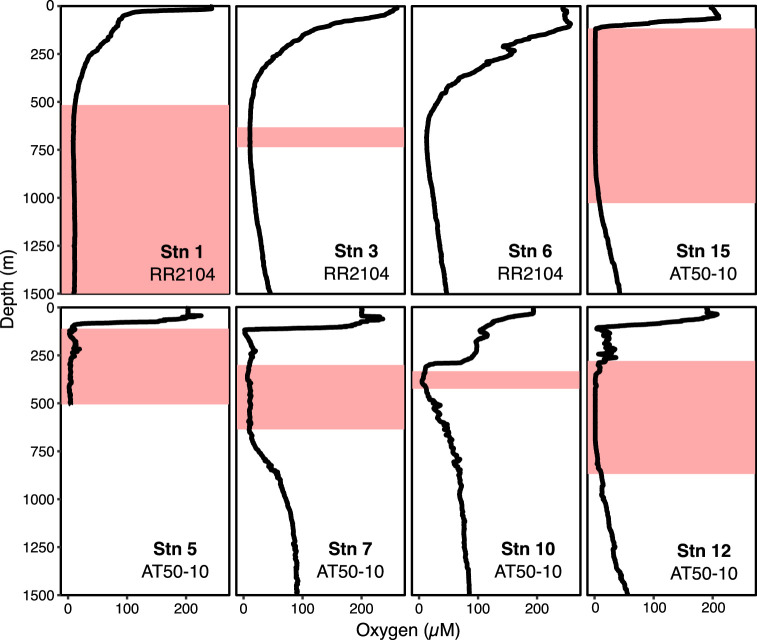
Oxygen concentration profiles of cruises RR2104 and AT50-10. The oxygen-deficient-zone (ODZ; O_2_ ≤ 10 µM) is shown by red shaded boxes. At Stn 6, O_2_ concentrations never dropped below 12 µM.

We sampled the eastern tropical and subtropical Pacific Ocean spanning 35° N to 10° S during two oceanographic sampling campaigns (Fig. 1a). Sampling stations (Stns) included regions of varying productivity, from productive coastal waters (Stns 1 and 3) to the equatorial upwelling region (Stn 10) and to oligotrophic offshore stations in the eastern tropical North Pacific (ETNP; Stns 6, 12 and 15) and South Pacific (ETSP; Stns 5 and 7). A thick oxygen-deficient zone (ODZ; O_2_ ≤ 10 µM) was present at most stations, with the narrowest ODZ of ~50 m found at the equatorial station (Extended Data Fig. 1). We observed pronounced subsurface chlorophyll maxima at all offshore stations (Fig. 1b) and secondary chlorophyll maxima at the two northernmost offshore ETNP stations (Stns 6 and 15). Ammonia oxidation rates ranged between 0.4 and 64 nM d^−1^, showed typical maxima close to the base of the euphotic zone and sharply declined below, except at the station closest to the coast (Stn 1), where ammonia oxidation rates remained comparably high in the mesopelagic zone (Fig. 1b).

**Fig. 2:**
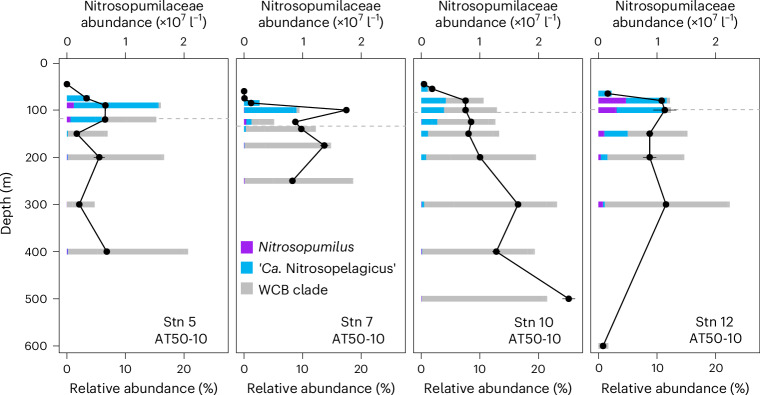
Nitrosopumilaceae abundances and community composition in the eastern tropical Pacific Ocean. Relative 16S rRNA gene abundances of different AOA clades are shown as a fraction of the total microbial community. *Ca*., *Candidatus*. Absolute Nitrosopumilaceae abundances were derived from quantitative PCR assays and are shown in black. Means and standard deviations from triplicate measurements are shown. The depth of the euphotic zone is indicated by the grey dashed horizontal lines.

Ammonia-oxidizing archaea (AOA) of the Nitrosopumilaceae family were the main ammonia oxidizers at all stations and depths, comprising up to 23% of the microbial community (Fig. 2). AOA abundances were low in surface waters and sharply increased to ~10^7^ cells l^−1^ close to the base of the euphotic zone, coincident with high ammonia oxidation rates and low to undetectable ammonium concentrations (Fig. 1b). Within the mesopelagic zone, AOA abundances remained relatively constant, except at anoxic depths (<1 µM O_2_), where their abundances declined substantially (for example, Stn 12 at 600 m depth). ‘*Candidatus* Nitrosopelagicus’ (water column A clade, WCA) was the dominant AOA genus in shallow waters, whereas members of the water column B (WCB) clade dominated below 100 m depth and were often the only ammonia oxidizers in the mesopelagic zone (Fig. 2). *Nitrosopumilus* 16S rRNA gene sequences were detected in shallow waters only at Stns 5 and 12. Abundances of ‘*Candidatus* Nitrosopelagicus’ showed a positive linear relationship with ammonia oxidation rates (*R*^2^ = 0.47, Supplementary Fig. 1a). In contrast, WCB clade abundances and ammonia oxidation rates were negatively related (*R*^2^ = 0.43, Supplementary Fig. 1b), suggesting that ‘*Candidatus* Nitrosopelagicus’ were the main contributors to ammonia oxidation at our study sites (Supplementary Results and Discussion).

## Specific inhibition of ammonia oxidation by phenylacetylene

**Fig. 3:**
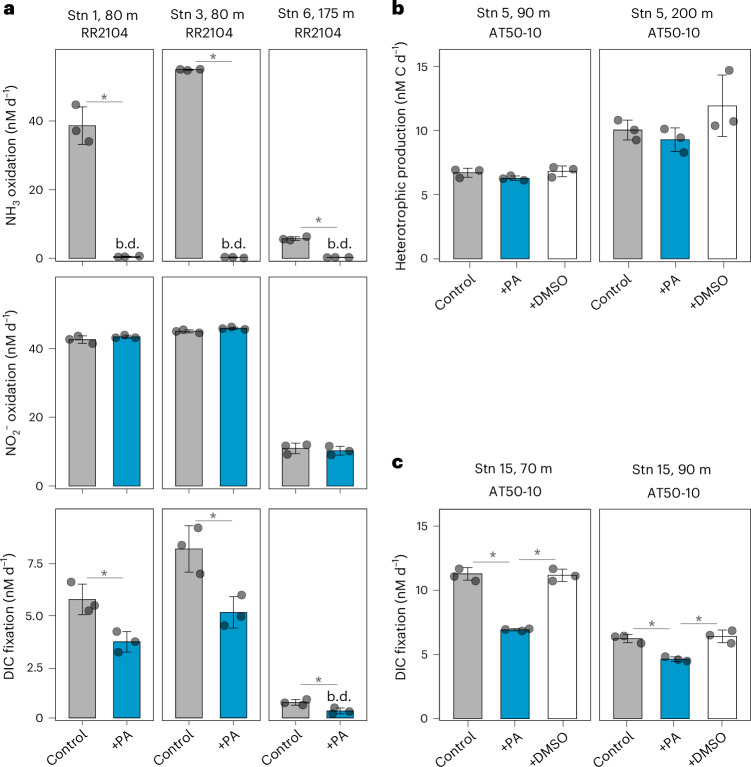
Effect of phenylacetylene on rates of microbial processes in ocean samples. **a**, Effect of 10 µM phenylacetylene additions (+PA) on ammonia (NH_3_) oxidation (top), nitrite (NO_2_^−^) oxidation (middle) and dark DIC fixation rates (bottom) at the indicated depths at Stns 1, 3 and 6 (left to right) during cruise RR2104. **b**,**c**, Comparison between additions of phenylacetylene (10 µM) dissolved in DMSO (0.01%) or DMSO alone (+DMSO) on microbial heterotrophic production (**b**) and dark DIC fixation rates (**c**) at the indicated depths at Stns 5 and 15 during cruise AT50-10, respectively. The mean and s.d. of three biological replicates are shown. *Significant difference between treatments (determined using one- or two-sided Student’s *t*-tests, *P* < 0.05). Details of the statistical analyses can be found in the Methods and Supplementary Table 1. Source data

A reliable method for specifically inhibiting the activities of ammonia oxidizers in ocean samples is required to isolate their contribution to dark DIC fixation. Phenylacetylene has previously been shown to inhibit cultures of terrestrial bacterial and archaeal ammonia oxidizers by irreversibly binding to the ammonia monooxygenase enzyme^31^, and is more practical for handling on oceanographic expeditions than the well-characterized inhibitor acetylene gas^32^. When applying inhibitors to complex microbial communities, it is crucial to evaluate and account for potential undesired effects on microbial community members other than the target organisms. We first determined the effective inhibitory concentration of phenylacetylene on the ammonia oxidation and DIC fixation activities of marine ammonia oxidizer cultures (≥5 µM, Supplementary Fig. 2 and Supplementary Results and Discussion). We also evaluated off-target effects of phenylacetylene on other members of the microbial community at select stations and depths. Phenylacetylene is known to inhibit other monooxygenase enzymes, including soluble and particulate methane monooxygenases^33^. However, methane monooxygenases are only inhibited at effective concentrations that are 10–100 times higher^33^ and relative abundances of putative methanotrophs were negligible at our study sites (≤0.04%, Supplementary Table 1). Phenylacetylene must be dissolved in dimethylsulfoxide (DMSO) due to its low solubility in water, which could affect microbial activity. Furthermore, phenylacetylene could be used as an energy source to support heterotrophic growth. When 10 µM phenylacetylene was added to whole seawater, ammonia oxidation was completely inhibited, while nitrite oxidation and microbial heterotrophic production rates did not significantly differ from those measured in control incubations without phenylacetylene (Fig. 3a,b). We also confirmed that DMSO alone had no significant effect on microbial heterotrophic production and dark DIC fixation during the time frame of our experiments (Fig. 3b,c). Consequently, phenylacetylene seems to be an effective specific inhibitor of ammonia oxidation activity in the ocean and can be used to infer the contributions of ammonia oxidizers to dark DIC fixation rates (‘ammonia-fuelled dark DIC fixation’).

## Total and ammonia-fuelled dark DIC fixation

**Fig. 4:**
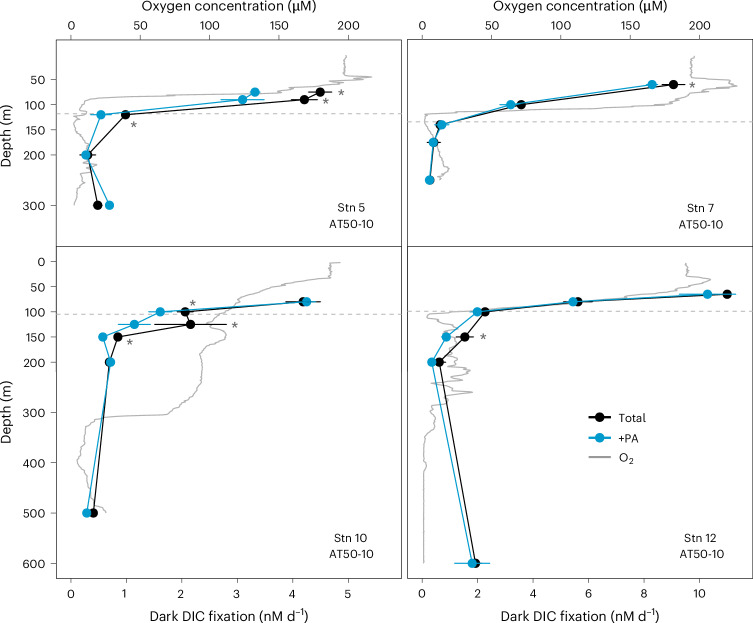
Depth-resolved dark DIC fixation rates in the eastern tropical Pacific Ocean. Total dark DIC fixation rates are depicted in black and dark DIC fixation rates after phenylacetylene addition (+PA) in blue. The mean and s.d. of three biological replicates are shown unless otherwise stated. Error bars were omitted for four experiments (PA-inhibited treatment of Stn 5 at 200 m depth, Stn 5 at 300 m depth and Stn 10 at 200 m depth and the control treatment of Stn 12 at 65 m depth); only duplicate measurements were available for these experiments due to issues during sample processing. Oxygen concentration profiles are depicted by grey solid lines and the depth of the euphotic zone by grey dashed lines. Note that the scales on the *x* axes are different for the stations in each column. *Significant difference between treatments (determined using one-sided Student’s *t*-tests, *P* < 0.05). Details of the statistical analyses can be found in the Methods and Supplementary Table 1. Source data

We measured total dark DIC fixation rates throughout the water column of four stations, spanning the ETNP, ETSP and equatorial Pacific (Fig. 4). Dark DIC fixation rates decreased with depth from the euphotic zone to the upper mesopelagic zone (defined here as below the euphotic zone and above 200 m depth), with the highest rates of ~11 nM d^−1^ observed at 65 m (Stn 12) and 70 m (Stn 15) depth in the ETNP (Figs. 3c and 4). DIC fixation rates in the lower mesopelagic zone (≥200 m depth) ranged between 0.3 and 1.9 nM d^−1^, with slightly increased rates in anoxic waters (O_2_ < 1 µM) at Stns 5 and 12, possibly due to the activities of anaerobic chemolithoautotrophs^34^. Depth-integrated dark DIC fixation rates ranged between 0.2 mmol C m^−2^ d^−1^ and 0.9 mmol C m^−2^ d^−1^, which is considerably lower than the range of rates in the North Atlantic (1.8–3.2 mmol C m^−2^ d^−1^; ref. ^13^). Organic matter export from the euphotic zone is estimated to be higher in the North Atlantic^35^ than in the eastern tropical Pacific Ocean, leading to higher overall productivity that could explain these observed differences.

We also determined the contribution of ammonia oxidizers to total dark DIC fixation in the euphotic and mesopelagic zones. When phenylacetylene was added to incubation bottles, dark DIC fixation rates significantly decreased on average by 24% (s.d. = 9%, *n* = 7) in the euphotic zone and by 41% (s.d. = 8%, *n* = 7) in the upper mesopelagic zone (Figs. 3 and 4). However, no statistically significant degree of inhibition was observed in the majority of incubation experiments (*n* = 14, Fig. 4), suggesting minor contributions of ammonia oxidizers to dark DIC fixation throughout most of the water column. Ammonia oxidizers are inhibited by light^36–38^ and nitrification rates in the euphotic zone are typically low under in situ light conditions^39–41^. While our incubations were performed in the dark, thereby excluding acute light inhibition, AOA abundances were particularly low in surface waters (Fig. 2), potentially explaining their low contribution to dark DIC fixation within the euphotic zone. Surprisingly, however, despite their high abundances in the lower mesopelagic zone, none of the incubation experiments showed significant inhibition after the addition of phenylacetylene (*n* = 7), indicating that ammonia oxidizers did not play a significant role in dark DIC fixation at these depths at the tested stations. We hypothesize that this could partly be due to the thick ODZ observed at most of the stations (Extended Data Fig. 1), and the reliance of ammonia oxidation on O_2_ availability^42^. Alternatively, WCB clade AOA might rely on other as-yet unknown metabolisms to support their high abundances in the deep ocean (Fig. 2 and Supplementary Results and Discussion).

Overall, ammonia oxidation could account for only 4–25% of the depth-integrated dark DIC fixation rates in the eastern tropical Pacific Ocean (Fig. 4). This implies that other microbial metabolisms contribute substantially to the cycling of inorganic C in the water column. Consequently, dark DIC fixation rates cannot be used to infer nitrification rates. In contrast, ammonia-fuelled dark DIC fixation rates could be inferred from ammonia oxidation rates if suitable conversion factors were available.

## DIC fixation yields of ammonia oxidizers in the ocean

**Fig. 5:**
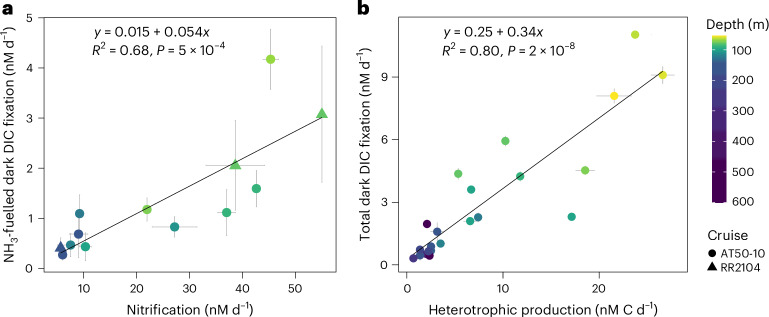
Relationships between dark DIC fixation, ammonia oxidation and heterotrophic production rates in the eastern tropical and subtropical Pacific Ocean. **a**, Linear relationship between ammonia oxidation rates and the fraction of dark DIC fixation inhibited by phenylacetylene. Ammonia-fuelled dark DIC fixation rates were calculated by subtracting phenylacetylene-inhibited measurements (*n* = 3) from non-inhibited measurements (*n* = 3); error bars show the propagated error using s.d. Only experiments for which significant differences between inhibited and non-inhibited treatments were observed were included (Supplementary Table 1). For ammonia oxidation rates, the mean and s.d. of three biological replicates are shown. **b**, Linear relationship between heterotrophic production and total dark DIC fixation rates. The mean and s.d. of three biological replicates are shown unless otherwise stated. Error bars were omitted from heterotrophic production measurements from Stn 12, for which only duplicate samples were taken. One data point (Stn 5, 200 m depth) was excluded due to unrealistically high heterotrophic production rates, possibly resulting from contamination or human error. The data points are colour coded by sample depth, with darker colours reflecting deeper depths. The two cruises are differentiated by symbol shape. Linear regression analysis was performed and the significance of the model fit (*P* < 0.05) was evaluated using *F*-statistic hypothesis testing. Details of the statistical analyses can be found in the Methods and Supplementary Table 1. Source data

We aimed to better constrain DIC fixation yields (moles of C fixed per mole of N oxidized) of ammonia oxidizers in the ocean to improve conversion factors for biogeochemical models. Ammonia oxidation and the fraction of dark DIC fixation inhibited by phenylacetylene (ammonia-fuelled dark DIC fixation) showed a strong positive linear relationship (*R*^2^ = 0.68, Fig. 5a). The average DIC fixation yield derived from the slope of the regression was ~0.05, which is considerably lower than those for cultures of ‘*Candidatus* Nitrosopelagicus brevis’ U25 and *Nitrosopumilus* sp. CCS1 isolated from the North Pacific Ocean (mean ± s.d. = 0.09 ± 0.01; ref. ^27^). The lower observed DIC fixation yields of AOA in the ocean might suggest a lower metabolic efficiency compared with ideal culture conditions. Our yield calculations, which relied on measuring ^15^N-ammonium-derived ammonia oxidation rates, did not take into consideration the possible preferential utilization of organic N sources (for example, urea) over ammonium^43^, which could theoretically lead to even lower environmental DIC fixation yields. Substantial urea utilization in the presence of experimentally added ammonium has been observed in the northwestern Pacific Ocean^44^; however, the potential impacts on ^15^N-ammonium-derived ammonia oxidation rates across different ocean regions are yet to be determined.

Although relatively low tracer additions (70–200 nM) were used in our study, the possible stimulation of ammonia oxidation is a relevant concern for rate measurements in the oligotrophic ocean and could lead to lower observed DIC fixation yields when measuring rates from separate incubation bottles (with DIC bottles not receiving corresponding additions of ammonium). This may have been the case on cruise AT50-10, where radioactivity safety protocols precluded combined rate measurements of nitrification and DIC fixation. In contrast, on cruise RR2104 ^13^C-labelled bicarbonate was used to measure DIC fixation rates in more productive waters, allowing us to combine both measurements within the same incubation bottle. Even when considering only samples from cruise RR2104, the average DIC fixation yields (0.054) were identical to those calculated from all data points. We therefore consider the DIC fixation yields of ammonia oxidizers in this study to be realistic environmental estimates for the eastern tropical and subtropical Pacific Ocean that can be used to inform theoretical models to better constrain the relationship between C and N fluxes in the dark ocean.

## Additional metabolisms contributing to dark DIC fixation

Ammonia is considered to be the primary energy source fuelling water column chemolithoautotrophy^18^ due to the higher molar ratio of N in marine organic matter relative to other potential energy sources, such as reduced sulfur (S) and iron^45,46^. Ammonia oxidizers supply nitrite (NO_2_^−^) to nitrite oxidizers, and the two steps in the nitrification process are typically tightly coupled^47^. Consequently, the inhibition of ammonia oxidation probably also results in the indirect inhibition of nitrite oxidation in the absence of an alternative NO_2_^−^ source. However, most of our stations were located in regions with pronounced ODZs, suggesting that NO_2_^−^ could also be supplied via nitrate reduction^48^. We assessed nitrite oxidation rates at selected depths (Supplementary Fig. 3) and used the average DIC fixation yield of cultured marine nitrite oxidizers (0.036, ref. ^27^) to estimate their potential contributions to dark DIC fixation. We estimate that ammonia and nitrite oxidizers together could, on average, account for 70% (s.d. = 22%, *n* = 10) of dark DIC fixation in the upper mesopelagic zone, but only 36% (s.d. = 10%, *n* = 5) within the euphotic zone. However, nitrite oxidizers in the open ocean are phylogenetically only distantly related to cultured representatives^20^ (Supplementary Fig. 4). Given the lower DIC fixation yields observed for ammonia oxidation in the environment compared with those of cultured ammonia oxidizers (Fig. 5a; ref. ^27^), the contributions of nitrite oxidizers to dark DIC fixation could be substantially lower than estimated here.

The genomic potential for chemolithoautotrophy fuelled by sulfur oxidation is widespread in the dark ocean^19,49,50^, and putative sulfur oxidizers were present at all stations (Supplementary Fig. 5, Supplementary Results and Discussion). The organic S content of sinking particulate organic matter is ~17 times lower than the N content^45^, suggesting a very limited supply of reduced S to the dark ocean. Even when considering the higher DIC fixation yields of sulfide oxidizers (0.15–0.35; refs. ^51,52^) compared with ammonia oxidizers (0.05; Fig. 5a), we estimate that sulfur-fuelled chemolithoautotrophy could amount to only one-third of that of ammonia-fuelled chemolithoautotrophy.

Heterotrophic microorganisms may also contribute to dark DIC fixation in the ocean^14,17,53^, particularly in surface waters where the uptake of DIC by different heterotrophic taxa has previously been shown to be high (40–200 nM C d^−1^; ref. ^54^). A compilation of >700 microbial heterotrophic production and dark DIC fixation measurements from the Atlantic and Pacific oceans indicated a positive linear relationship between both processes (*R*^2^ = 0.45; ref. ^14^). We found a similar, yet stronger, relationship between microbial heterotrophic production and total dark DIC fixation in the eastern tropical Pacific Ocean (*R*^2^ = 0.80; Fig. 5b). The sparse available data on heterotrophic DIC fixation suggest that 1–10% of C in bacterial biomass is derived from DIC assimilation^55–57^. When we assumed that heterotrophs incorporate 10% of their C as DIC, we could explain on average 30% (s.d. = 15%, *n* = 22) of the dark DIC fixation rates in the epi- and upper mesopelagic zones.

## Implications for the dark ocean’s C budget

Our data confirm that ammonia oxidation is an important process in the upper mesopelagic zone, but we show that it contributes a much lower percentage of dark DIC fixation than previously assumed, amounting to a maximum of 25% of the depth-integrated rates in the eastern tropical Pacific Ocean. When including high-end estimates of nitrite- and sulfur-fuelled chemolithoautotrophy and heterotrophic DIC fixation, 36–111% of the depth-integrated dark DIC fixation rates could be explained (Table 1). Discrepancies remain, particularly within the euphotic zone, where the contributions of ammonia and nitrite oxidizers to total dark DIC fixation are comparably small, and in the lower mesopelagic zone (≥200 m depth), where the flux of particulate organic matter from the surface is often insufficient to provide the energy sources required to sustain measured dark DIC fixation rates at depth. Constraining the contributions of sulfur oxidizers and heterotrophs will be crucial to reconcile these observed discrepancies.

**Table 1:** Contributions of different metabolisms to depth-integrated dark DIC fixation rates in the eastern tropical Pacific Ocean

Depth-integrated rates	Stn 5	Stn 7	Stn 10	Stn 12
Total measured dark DIC fixation (µmol m^−2^ d^−1^)	234	382	348	860
Ammonia-fuelled chemoautotrophy (µmol m^−2^ d^−1^)	58 (25%)	15 (4%)	48 (13%)	33 (4%)
Nitrite-fuelled chemoautotrophy (µmol m^−2^ d^−1^)	82 (35%)	76 (20%)	23 (6%)	57 (7%)
Sulfur-fuelled chemoautotrophy (µmol m^−2^ d^−1^)	24 (10%)	6 (2%)	20 (5%)	14 (2%)
Heterotrophic DIC fixation (µmol m^−2^ d^−1^)	95 (41%)	99 (26%)	107 (31%)	203 (24%)
All metabolisms (µmol m^−2^ d^−1^)	259 (111%)	196 (51%)	198 (56%)	308 (36%)

Total and ammonia-fuelled dark DIC fixation rates were measured directly, whereas the contributions of other metabolisms were estimated. Estimates of nitrite-fuelled chemoautotrophy were based on measured nitrite oxidation rates and the average DIC fixation yield of nitrite oxidizer cultures (0.036; ref. ^27^). Heterotrophic DIC fixation rates were estimated as 10% of the measured heterotrophic production rates. Sulfur-fuelled chemoautotrophy was estimated from ammonia-fuelled chemoautotrophy values based on differences in the N:S stoichiometry of particulate organic matter (17:1; ref. ^45^) and differences between the DIC fixation yields of ammonia oxidizer (Fig. 5a) and sulfide oxidizer cultures (0.35; ref. ^51^). Percentages in parentheses show the estimated contribution of each metabolism to measured total dark DIC fixation rates.

Our results improve our understanding of microbial processes in the dark ocean and offer insights into the main energy sources fuelling dark DIC fixation. Our findings have broad implications as they provide critical conversion factors for biogeochemical models of the mesopelagic C budget, which are essential to better predict the impact of future climate scenarios on the biological sequestration of C in the ocean.

## Methods

### Cruise track and dissolved nutrient analyses

Water samples were collected during two oceanographic cruises in the eastern tropical and subtropical Pacific Ocean aboard the R/V *Roger Revelle* (cruise RR2104: 15–29 June 2021) and the R/V *Atlantis* (CliOMZ, cruise AT50-10: 2 May–9 June 2023) at a total of eight stations spanning 35° N to 10° S (Fig. 1a). On both cruises, hydrographic data were collected with an SBE-911plus conductivity, temperature, depth (CTD) sensor package (SeaBird Scientific) that was also equipped with a fluorometer (ECO, Seabird Scientific) and a Clark-type electrode oxygen sensor (SBE 43, SeaBird Scientific). Discrete water samples were collected using a rosette sampler equipped with 24 10 l Niskin bottles.

Ammonium (NH_4_^+^) concentrations were measured on board from unfiltered 40 ml seawater samples using the *o*-phthaldialdehyde derivatization method^59^ with the modifications suggested in ref. ^60^ on an Aquafluor 8000 handheld fluorometer (Turner Designs). NH_4_^+^ standards (30–300 nM) were freshly prepared for each station using deep water (>500 m), which consistently had a lower blank than ultrapure water. Samples for nitrite and nitrate concentration measurements were syringe-filtered (0.22 µm, Sterivex) and stored at −20 °C before concentrations were determined by Cd reduction coupled to colorimetric detection via the Griess assay on a QuikChem 8500 Series 2 flow injection analysis system (Lachat Instruments)^61^. The depth of the euphotic zone was estimated as the depth below the chlorophyll *a* maximum, where chlorophyll *a* fluorescence was 10% of the maximum fluorescence value^62^, roughly corresponding to 0.1% of the surface photosynthetically active radiation. CTD casts were rarely done during midday and the photosynthetically active radiation values could therefore not be consistently used to measure the depth of the euphotic zone.

### Nitrification rates

Nitrification rates (both ammonia and nitrite oxidation rates) were determined using ^15^N isotope tracer methods. For depths where O_2_ concentrations were >20 µM, incubations were conducted in 250 ml or 1 l polycarbonate bottles (Nalgene). For depths where O_2_ concentrations were ≤20 µM, incubations were either conducted in 500 ml Tedlar bags (Restek) or 120 ml glass serum bottles following procedures to prevent O_2_ contamination (Supplementary Methods). For each incubation depth, three bottles or bags were filled and spiked with [^15^N]-tracer (99 atm% ^15^NH_4_Cl or 98 atm% Na^15^NO_2_^−^, Cambridge Isotope Laboratories) to a final label concentration of 70–200 nM, depending on the depth and productivity region the sample was collected from. Across all incubation experiments, the ^15^N-labelling percentage varied between 5% and 99%, with an average of 88% (s.d. = 21%).

Nitrification rates were determined throughout the water column, targeting depths in the middle of the euphotic zone, the upper and lower mesopelagic zone. Nitrite oxidation rates were measured during cruise AT50-10 at a lower resolution than ammonia oxidation rates (Supplementary Fig. 3). Incubations were conducted in the dark in temperature-controlled incubators within ±1.5 °C of the in situ temperature. At time points of 0, 12 and 24 h, 50 ml samples were drawn from each bottle or bag, filtered using 0.2 µm syringes into a 20 ml HDPE bottle and frozen at −20 °C. Frozen samples were transported to the laboratory, thawed and prepared for δ^15^N_NO2+NO3_ analysis using the denitrifier method^63^. For nitrite oxidation rate samples, the added ^15^NO_2_^−^ tracer was removed by adding sulfamic acid and subsequently neutralizing with NaOH before sample preparation^64^. Samples were analysed on a custom purge and trap system interfaced with a Thermo Delta Plus XP isotope ratio mass spectrometer^65^. The δ^15^N_NOx_ values were calibrated against NO_3_^−^ isotope reference materials USGS 32, USGS 34 and USGS 35, analysed in parallel. Ammonia and nitrite oxidation rates were calculated using the basic equations of Dugdale and Goering^66^. Detection limits for ammonia oxidation and nitrite oxidation rates were calculated as described in ref. ^34^ and ranged from 0.75 nM d^−1^ during cruise AT50-10 to 1.42 nM d^−1^ during cruise RR2104.

### Dark DIC fixation rates

During cruise RR2104, DIC fixation was measured via the incorporation of [^13^C]bicarbonate. Water was sampled from Niskin bottles into 1 l polycarbonate bottles (Nalgene). For each station, the depth of the expected nitrification maximum was sampled, and 12 bottles were filled, to which [^13^C]bicarbonate and either [^15^N]ammonium or [^15^N]nitrite were added (see ‘Nitrification rates’). [^13^C]bicarbonate tracer concentrations were made as 20% additions of the ambient bicarbonate pool. Phenylacetylene (10 µM) dissolved in DMSO (0.01%) was added to six of the incubation bottles to inhibit ammonia oxidation activities, and all bottles were incubated in the dark in temperature-controlled incubators (within ±1.5 °C of the in situ temperature). After 24 h of incubation, samples were filtered onto pre-combusted glass fibre filters (GF75, 25 mm, Advantec) and frozen at −80 °C until further processing was conducted. Filters were acidified by acid fumigation to remove inorganic carbonates and dried at 60 °C for at least 24 h. The δ^13^C of particulate organic C was measured with a Finnigan Delta-Plus Advantage isotope ratio mass spectrometer (Thermo Scientific) coupled with an elemental analyser (Costech EAS). Acetanilide reference standards were run at the beginning of each set of 35 samples and tested every 5 samples within each set. The instrument precision, determined using replicate analyses of l-glutamic acid USGS 40, was ±0.1‰ for ^13^C. The δ^13^C of DIC in water samples was measured with a GasBench II system interfaced with a Delta V Plus isotope ratio mass spectrometer (Thermo Scientific) at the University of California Davis Stable Isotope Facility. DIC fixation rates were calculated as the absolute amount of ^13^C incorporated into particulate organic C above background levels and corrected for the in situ DIC concentration as described in ref. ^67^. The detection limit, calculated as the rate required to produce an isotopic change more than three times the standard deviation of *T*_0_ (starting time) measurements, was 0.73 nM d^−1^ at the offshore station (Stn 6) and 1.47 nM d^−1^ at the two coastal stations (Stns 1 and 3).

During cruise AT50-10, DIC fixation was measured via the incorporation of [^14^C]bicarbonate^68^. For depths where O_2_ concentrations were >20 µM, water was sampled into 1 l polycarbonate bottles (Nalgene) and later dispersed into 40 ml glass vials with teflon coated silicon septa (TOC-certified, Fisher Scientific). We initially used 50 ml conical centrifuge tubes (Fisher Scientific) for oxic incubations, however, we noticed that DIC fixation rates were highly variable and much higher in plastic than in glass tubes (Supplementary Results and Discussion and Supplementary Fig. 6). Consequently, none of the data obtained from measurements in plastic tubes could be used for further analysis, and we switched to glass tubes by Stn 5. For depths where in situ O_2_ concentrations were ≤ 20 µM, water was sampled directly from the Niskin bottle into 60 ml glass serum bottles following procedures to avoid O_2_ contamination (Supplementary Methods).

For each incubation depth, seven or eight replicate bottles were filled and spiked with 50 µCi [^14^C]bicarbonate (specific activity 56 mCi mmol^−1^ or 2.072 × 10^9^ Bq mmol^−1^; PerkinElmer). To three bottles, 10 µM phenylacetylene dissolved in DMSO (0.01% final concentration) was added to inhibit ammonia oxidation activities. One or two bottles served as killed controls to which formaldehyde (3% vol/vol) was added at the start of incubation. Bottles were incubated in the dark within *±*1.5 °C of the in situ temperature and handled under red light to prevent ^14^C assimilation by phytoplankton. Live incubations were terminated after 24–72 h by adding formaldehyde (3% vol/vol), and after 30–60 min, samples were filtered onto 0.2-μm polycarbonate filters (GTTP, 25 mm, Millipore) and rinsed with 10 ml of artificial seawater. The filters were transferred to scintillation vials and 10 ml of scintillation cocktail (Ultima Gold; PerkinElmer) was added. Samples were shaken for approximately 30 s and placed in the dark for at least 24 h before counting the disintegrations per minute (DPM) in a scintillation counter (PerkinElmer Tri-Carb 2910 TR) for 15 min. Total radioactivity measurements were performed to verify the added [^14^C]bicarbonate concentrations by pipetting 100 μl of unfiltered sample into scintillation vials containing 400 μl beta-phenylethylamine, and samples were immediately measured after the addition of the scintillation cocktail. DIC fixation rates were calculated by subtracting the DPM of the blanks from the DPM of the samples, converted into C fixed over time and corrected for the in situ DIC concentration^27^. The detection limit, calculated as the rate required to produce a change in DPM more than three times the standard deviation of *T*_0_ measurements, was 0.18 nM d^−1^ below the euphotic zone and 0.37 nM d^−1^ within the euphotic zone.

Owing to the different methodologies used on both cruises to infer DIC fixation rates, we compared rates obtained from [^13^C]bicarbonate and [^14^C]bicarbonate incorporation on a culture of the ammonia-oxidizing archaeon *N. adriaticus* NF5 and found good agreement between the two methods (Supplementary Results and Discussion and Supplementary Fig. 7a). We further confirmed that [^14^C]bicarbonate incorporation rates were linear over the length of the incubation time (up to 3 d; Supplementary Results and Discussion and Supplementary Fig. 7b).

### Leucine incorporation rates

Microbial heterotrophic production rates were measured via the incorporation of [^3^H]leucine into microbial biomass using a modified version of the microcentrifuge method^69^. Briefly, [^3^H]leucine (specific activity 44.9 Ci mmol^−1^; PerkinElmer) was added to 1.6 ml of sample at a final concentration of 20 nM and incubated for 2–3 h at the in situ temperature. For each depth and station, triplicate live incubations and one killed control incubation (to which 100 µl trichloroacetic acid (TCA) was added immediately) were carried out. Incubations were terminated by adding 100 µl cold 100% TCA and stored at 4 °C until extraction. Proteins were extracted with 5% TCA and rinsed with 80% ethanol following a series of centrifugation steps (16,000 *g*, 7 min)^70^, before 1.5 ml scintillation cocktail (Ultima Gold, PerkinElmer) was added to each tube and DPM were measured on a scintillation counter (PerkinElmer Tri-Carb 2910 TR) for 2 min. [^3^H]leucine incorporation rates were converted to units of C using a conversion factor of 1.5 kg C per mol leucine incorporated^71^. To test the effects of phenylacetylene (10 µM) and DMSO (0.01%) on microbial heterotrophic production, [^3^H]leucine was added to a final concentration of 60 nM and incubated for 24 h. The different incubation time and substrate concentration were selected to monitor potential inhibition over a longer time frame. All other steps followed the procedures described above. The detection limit ranged from 0.24 to 0.56 nM d^−1^, calculated in the same way as for the DIC fixation rates described above.

### Quantitative PCR and metagenome analysis

Seawater was collected in acid-washed 4 l polycarbonate bottles and the biomass was sequentially filtered onto 5-µm (25 mm, PETE, Sterlitech Corporation) and 0.22-µm (25 mm, Supor PES, Pall) pore-size membrane filters, which were subsequently frozen at −80 °C until extraction. DNA was extracted from 0.22-µm filters using the Qiagen DNeasy Blood & Tissue kit with modifications for the use of filters described in ref. ^72^.

Quantitative PCR assays were conducted using group-specific assays for the 16S RNA gene of marine AOA and NOB of the Nitrospinaceae family following established protocols and thermocycling conditions^72,73^. We modified the previously published AOA primers^73^ to increase the coverage of members of the *Nitrosopumilus* genus (MGI_F: 5′-GTC TAC CAG AAC ACG TYC-3′; MGI_R: 5′-WGG CGT TGA CTC CAA TTG-3′). Gene copies were quantified on a CFX96 qPCR machine (Bio-Rad) with SYBR Green chemistry. All samples were run in triplicate against a standard curve spanning approximately 10^1^–10^6^ template copies. Linearized plasmids containing cloned inserts of the target genes (TOPO pCR4 vector, Invitrogen) were used as standards^74^. Fresh standard dilutions were made from frozen stocks for each day of analysis. The efficiencies of the assays were 99.5–100.5%.

DNA libraries were prepared by the UC Davis Genome Center and sequenced on two flow cells of the AVITI sequencer (Element Biosciences) run with paired-end 300-bp reads. Resulting reads were trimmed and quality-filtered and internal standards were removed using the default settings of Bowtie 2 (v. 2.5.2)^75^ and Trimmomatic (v. 0.39)^76^. Microbial community compositions were subsequently assessed by mapping reads to the SILVA SSU rRNA reference database (v. 138.2) using the default settings of phyloFLASH (v 3.4.2)^77^. Detailed descriptions of sequencing methods and bioinformatic analyses are provided in the Supplementary Methods.

### Statistical analyses

The normal distribution of the data was evaluated with a Shapiro–Wilk Test. A one-sided Student’s *t*-test was used to test for inhibition after phenylacetylene addition, and a two-sided Student’s *t*-test was used to test for both inhibition and stimulation after phenylacetylene or DMSO additions. Bonferroni corrections were applied when multiple comparisons were made. Statistical analysis was conducted using the stats package in the R (v. 4.1.2) software environment^78^. Linear regression analysis was used to describe the relationships between DIC fixation, ammonia oxidation and heterotrophic production using the ‘lm’ method (default parameters) within the stats package. Test statistics can be found in Supplementary Table 1.

### Data visualization

All plots were generated in the R (v. 4.1.2) software environment^78^ using the ggplot2 package^79^. The station map was generated with the R package ggOceanMaps^80^ using the ETOPO 2022 bathymetry data^58^.

## Online content

Any methods, additional references, Nature Portfolio reporting summaries, source data, extended data, supplementary information, acknowledgements, peer review information; details of author contributions and competing interests; and statements of data and code availability are available at 10.1038/s41561-025-01798-x.

## Supplementary information

Supplementary Information Supplementary Figs. 1–7, Methods, Results and Discussion and references.

Supplementary Table 1 Microbial community composition, MAG analyses and test statistics.

## Source data

Source Data Fig. 3 Statistical source data.

Source Data Fig. 4 Statistical source data.

Source Data Fig. 5 Statistical source data.

## Data Availability

Hydrographic data from cruises RR2104 and AT50-10 are available on the Rolling Deck to Repository (R2R) webpage (https://www.rvdata.us) under the respective cruise names. Metagenome sequencing data generated during this study are available in the NCBI Sequence Read Archive under BioProject number PRJNA1179712 (https://www.ncbi.nlm.nih.gov/bioproject/?term=PRJNA1179712); raw metagenomic sequences are available under accession numbers SRR31156201–SRR31156268 and SRR31341463–SRR31341480. PhyloFLASH analyses and a description of metagenome-assembled-genomes (MAGs) analysed in this study are provided in Supplementary Table 1. Environmental data are available from the Biological and Chemical Oceanography Data Management Office (BCO-DMO) repository: dark DIC fixation rate data are available at 10.26008/1912/bco-dmo.948396.4 (ref. ^81^), ammonia and nitrite oxidation rate data at 10.26008/1912/bco-dmo.954872.1 (ref. ^82^), heterotrophic production rate data at 10.26008/1912/bco-dmo.948411.1 (ref. ^83^), nutrient profile data at 10.26008/1912/bco-dmo.948503.1 (ref. ^84^) and nitrifier abundance data at 10.26008/1912/bco-dmo.970545.1 (ref. ^85^). Source data are provided with this paper.
